# Recessive Mutation in *FAM83G* Associated with Palmoplantar Keratoderma and Exuberant Scalp Hair

**DOI:** 10.1016/j.jid.2017.10.031

**Published:** 2018-04

**Authors:** Thiviyani Maruthappu, Lisa A. McGinty, Diana C. Blaydon, Benjamin Fell, Arto Määttä, Rebecca Duit, Tim Hawkins, Kristin M. Braun, Michael A. Simpson, Edel A. O’Toole, David P. Kelsell

**Affiliations:** 1 Blizard Institute, Barts and the London School of Medicine and Dentistry, Queen Mary University of London, London, UK; 2 Department of Biosciences, Durham University, Durham, UK; 3 Division of Genetics and Molecular Medicine, King's College London, London, UK

To the Editor

Palmoplantar keratodermas are a heterogeneous group of disorders characterized by abnormal thickening of the volar epidermis (Blaydon and Kelsell, 2014, Maruthappu et al., 2014). A subset of palmoplantar keratodermas are associated with syndromes linked to other cutaneous features (Betz et al., 2012) and also noncutaneous conditions such as hearing loss, cardiomyopathy, and esophageal cancer (Blaydon Diana et al., 2012, Kelsell et al., 2001). Palmoplantar keratodermas specifically associated with defects in hair development include the desmosomal disorders linked to phenotypes such as woolly hair and alopecia (Brooke et al., 2012).

Two adult siblings from a consanguineous family of Pakistani origin, whose parents were first cousins, presented with an autosomal recessively inherited palmoplantar keratoderma, leukonychia, and exuberant curly scalp hair (Figure 1a). Both affected individuals described the progressive development of yellowish thickened scaly skin affecting the palms and soles since 2 years of age, and toenail dystrophy in their teenage years. Examination revealed marked diffuse, verrucous hyperkeratosis with deep fissuring affecting the soles (Figure 1a) and to a lesser extent, the palms. There was no evidence of transgradiens. The toenails were dystrophic with onycholysis and leukonychia was also present, most evident in the finger nails. Onychomycosis was excluded by negative fungal culture. No abnormalities of teeth or sweating were identified. The siblings also described having extremely thick, rapidly growing curly scalp hair since childhood, but without excessive hair growth elsewhere. Neither parent had a similar hair or skin phenotype, and they had no other offspring. Clinical photographs were obtained, and written consent was provided by patients for their publication. Blood samples were collected after written informed consent in adherence with the Declaration of Helsinki principles and approval of the East London and City Health Authority. Whole-exome capture from both siblings was performed using SeqCap EZ Human Exome Library v2.0 (Roche NimbleGen, Madison, WI) and sequenced with 100-bp paired-end reads on the HiSeq 2000 platform (Illumina, San Diego, CA). Resulting reads were mapped to the hg18 human reference genome using the Novoalign alignment tool (Novocraft Technologies Sdn Bhd, Selangor, Malaysia). Sequence variants were called with SAMtools and annotated with ANNOVAR (Wang et al., 2010).

**Figure 1:**
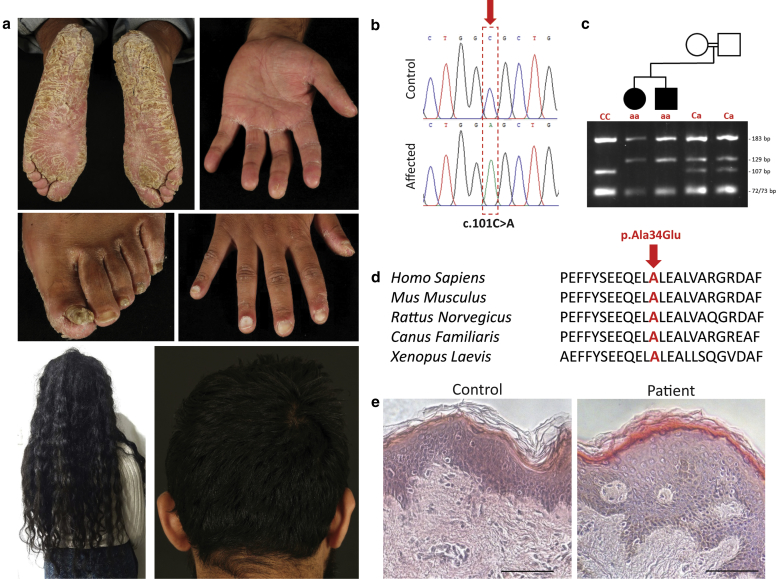
**A missense variant in FAM83G is associated with autosomal recessive palmoplantar keratoderma and exuberant scalp hair.** (**a**) Two siblings presented with diffuse palmoplantar keratoderma, displaying marked fissuring particularly affecting the soles, in combination with dystrophic toenails, leukonychia, and thick, bushy scalp hair. (**b**) Homozygous C to A mutation, c.101C>A, in *FAM83G* identified by exome sequencing was confirmed by Sanger sequencing. (**c**) Segregation of the mutation within the family was confirmed by restriction fragment length polymorphism analysis (genotypes in red). (**d**) The mutation c.101A is predicted to result in the missense variant, p.A34E, in the FAM83G protein affecting an alanine residue (indicated in red) that is evolutionary conserved across vertebrates. (**e**) Hematoxylin and eosin staining of affected skin reveals acanthosis.

Given the history of parental relatedness, the variants were filtered for homozygous changes shared by the two affected individuals, of which 83 homozygous variants reported either as novel or with an Exome Variant Server (EVS) (NHLBI GO Exome Sequencing Project, Seattle, WA) estimated frequency of less than 0.01 were selected as potential candidates. These candidates included a homozygous C to A transversion, c.C101A, in exon 2 of *FAM83G* (NM_001039999), a gene reported to be mutated in hereditary footpad hyperkeratosis in Kromfohrländer and Irish terrier dog breeds, which presents with fissuring hyperkeratosis of the paws and a bushy coat (Drogemuller et al., 2014, Sayyab et al., 2016). Furthermore, the bushy hair phenotype of the “woolly” mice (*wly* mouse) has been linked to a 995-bp deletion in *fam83g* (Radden et al., 2013). Therefore, the variant in *FAM83G* presented as an obvious candidate for further analysis.

The homozygous c.C101A variant in *FAM83G* was confirmed by Sanger sequencing in both siblings, and both parents were heterozygous carriers (primer sequences: FAM83G-F: 5′ CCGGGCTCATCAGGTCTTT 3′ and FAM83G-2R: 5′ GAGCGGTCCGACTTCTGG 3′; Figure 1b). The c.101C>A mutation results in loss of a Cac8I restriction endonuclease consensus site and segregation of the mutation with the condition was confirmed by restriction fragment length polymorphism analysis (Figure 1c). The mutation c.C101A is predicted to change an evolutionary conserved alanine to glutamate (p.A34E) in the protein FAM83G. This missense mutation was absent from the database of Single Nucleotide Polymorphisms (dbSNP), ExAC, 1000 genomes, gnomAD, and EVS. It is predicted to be deleterious to the protein structure (Polyphen2 score 1.00, SIFT score 0.00). This residue is conserved across vertebrates (Figure 1c) as well as between members of the FAM83 family of proteins. The FAM83A-H family is characterized by the presence of a conserved N-terminal domain of unknown function: DUFI 669. Like the missense variant, p.A34E, reported in this study, the missense variant, p.R52P, underlying hereditary footpad hyperkeratosis in dogs is also located in the DUFI 669 domain. FAM83G expression is specifically enriched (>fivefold) in the skin compared with 26 other tissue types (Edqvist et al., 2015). FAM83G has been implicated as a regulator of BMP signaling (Vogt et al., 2014).

A skin biopsy obtained from one of the siblings revealed acanthosis of the epidermis (Figure 1e), whereas immunohistochemistry demonstrated a marked reduction in FAM83G (Abcam ab121750, Cambridge, UK) expression compared with control skin (Figure 2a). Upregulation of Ki67 (Abcam ab15580) was evident, and keratin 14 expression (Abcam LL001) was not restricted to the basal layer suggesting dysregulated proliferation.

**Figure 2:**
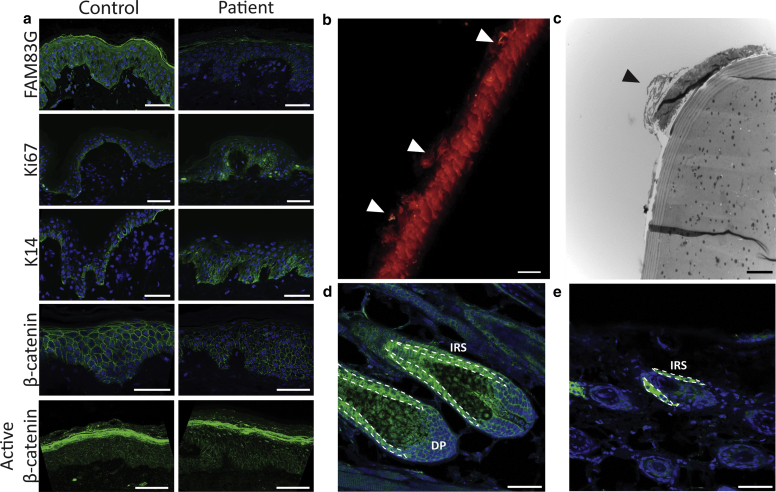
**Immunohistochemical analysis of patient skin reveals a hyperproliferative phenotype with hair shaft abnormalities.** (**a**) Patient epidermis displays reduced FAM83G expression, marked upregulation of Ki67 and keratin 14 (K14), and increased active nuclear β-catenin expression compared with healthy control (scale bar: 50 μm). (**b**) Confocal imaging of the hair shaft from the affected sibling shows membrane-bound deposits on the outer cuticle of the hair shaft (scale bar: 25 μm). (**c**) Transmission electron microscopy of a cross section of the affected siblings’ hair shaft shows the regions of poorly differentiated cells (scale bar: 4 μm). (**d**) FAM83G is expressed within the murine hair follicle during anagen and (**e**) in catagen/early telogen (scale bar: 50 μm). DP, dermal papilla; IRS, inner root sheath.

As keratinocyte and hair growth development has been linked to Wnt signaling, immunohistochemical staining of β-catenin (BD Transduction Labs 610153, Oxford, UK) was performed and increased levels of active β-catenin were seen within cell nuclei of the patient epidermis compared with control (Merck Millipore 05-655 Clone 8E7, Watford, UK) (Figure 2a and b). As nuclear translocation of β-catenin is induced by activation of Wnt signaling (Greco et al., 2009), FAM83G may be a repressor of Wnt signaling and proliferation.

To investigate the hair phenotype, hair from one sibling and both parents were obtained. Confocal imaging showed membrane-bound deposits on the outer cuticle of the hair shaft present in the affected sibling but absent in both parents (Figure 2b). Transmission electron microscopy revealed consistent regions of poorly differentiated cuticle cells possibly deriving from the inner root sheath (Figure 2c). FAM83G expression within hair follicles from the back skin of control mice was investigated. FAM83G was found to be expressed during the anagen growth phase (P28) (Figure 2d) and exhibited high levels of staining intensity at the inner root sheath and in the dermal papilla. The expression of FAM83G appeared to be maintained in catagen/early telogen (P42), within the inner root sheath (Figure 2e). FAM83G has previously been identified as a differentially expressed gene in adult mouse hair bulge stem cells compared with dermal papilla cells during telogen (P56) (Greco et al., 2009).

Our findings highlight a key role for FAM83G in the homeostasis of the palmoplantar epidermis and hair. Dampened Wnt/β-catenin signaling is a feature of several common forms of hair loss, including alopecia areata and androgenetic alopecia. Further studies of FAM83G, in particular regarding its role in hair biology and the hair cycle, may support targeting FAM83G and associated pathways in scalp hair disorders.

## ORCID

David P. Kelsell: http://orcid.org/0000-0002-9910-7144

## Conflict of Interest

The authors state no conflict of interest.
